# G-Quadruplexes in Repeat Expansion Disorders

**DOI:** 10.3390/ijms24032375

**Published:** 2023-01-25

**Authors:** Ye Teng, Ming Zhu, Zhidong Qiu

**Affiliations:** School of Pharmacy, Changchun University of Chinese Medicine, 1035 Boshuo Road, Changchun 130117, China

**Keywords:** G-quadruplex, repeat expansion disorder, neurodegeneration, RNA foci

## Abstract

The repeat expansions are the main genetic cause of various neurodegeneration diseases. More than ten kinds of repeat sequences with different lengths, locations, and structures have been confirmed in the past two decades. G-rich repeat sequences, such as CGG and GGGGCC, are reported to form functional G-quadruplexes, participating in many important bioprocesses. In this review, we conducted an overview concerning the contribution of G-quadruplex in repeat expansion disorders and summarized related mechanisms in current pathological studies, including the increasing genetic instabilities in replication and transcription, the toxic RNA foci formed in neurons, and the loss/gain function of proteins and peptides. Furthermore, novel strategies targeting G-quadruplex repeats were developed based on the understanding of disease mechanism. Small molecules and proteins binding to G-quadruplex in repeat expansions were investigated to protect neurons from dysfunction and delay the progression of neurodegeneration. In addition, the effects of environment on the stability of G-quadruplex were discussed, which might be critical factors in the pathological study of repeat expansion disorders.

## 1. Introduction

Neurological degenerative diseases, which are devastating diseases without efficient treatments, have attracted the interest of many researchers and progressed rapidly in the past decades. Dementias have become a globally problem as one of the World Health Organization’s (WHO) top 10 causes of death in 2016 [1]. The effective treatment of neurodegenerative diseases is an urgent need. However, the disease mechanism of neurodegeneration is still not fully understood. In neurodegeneration, the structures or functions of neurons are damaged, even resulting in the death of neuronal cells. Unlike continuously dividing cells, neurons are terminally differentiated cells whose regeneration, especially in the central nervous system, is greatly inhibited by the environment. Though new neurons were found to continue generating in the hippocampus [2], the increasing neurogenesis might induce forgetting [3]. On the other hand, the studies of neural stem cells demonstrated that they were not sufficient to repair the damage caused by neuronal apoptosis [4]. Delaying and reducing cell apoptosis in progressive neurodegeneration, which provides the condition for the self-repair of nervous system, is very important in the treatment of neurological degenerative diseases. Further understanding of the mechanism of neurodegeneration and its development is required.

With the analysis of human genomes, many human diseases, including both familial and sporadic cases, are found to generate from genetic mutations. The investigations at genetic level deepen our understanding of disease pathogenesis and provide new inspirations in the development of novel therapies. Repeat expansion is one of pathogenic gene mutations, which has been demonstrated to cause many neurodegeneration diseases [5,6,7,8,9,10], such as amyotrophic lateral sclerosis (ALS) [11,12], frontotemporal dementia (FTD) [11,12], spinal and bulbar muscular atrophy (SBMA) [13], spinocerebellar ataxia (SCA) [14,15,16,17,18], Parkinson’s disease (PD) [19], and Huntington’s disease (HD) [20,21,22]. The repetitive sequences are usually with high G/C contents, and their lengths are variable through generations, which are tens to thousands of times larger than healthy people. The dynamic genetic mutations are kept ongoing within tissues and generations, and the extensive size variabilities of repeat expansions were observed in different patients and even different tissues of the same patient [23]. As a result, these repeat expansions induced neurodegeneration diseases, which showed various phenotypes. Furthermore, the repeat size was reported correlated with not only the onset age, but also the methylation of DNA and the regulation of transcription [24]. Exploring the pathogenesis of repeat expansions will help us deeply understand the occurrence of neurodegeneration and effectively delay its development.

An important feature of repeat expansion is the unique secondary structure. The repeat sequences can form multiple non-canonical structures, for example, hairpins, triplexes, and quadruplexes, increasing the gene instability and affecting gene expression processes including DNA replication, repair, and transcription. As these repeats usually have high G/C contents, G-quadruplex becomes one of the common non-canonical structures in repeat expansions. G-quadruplex is composed of π–π stacked G-quartets, where guanines interact with each other through Hoogsteen base pairing to form plane structures. Both DNA and RNA G-quadruplexes have been demonstrated to play important roles in many bioprocesses. DNA G-quadruplexes widely exist in the regulatory regions of genome and participate the regulation of replication and transcription. Similarly, the formation of G-quadruplex in RNA also modulates the process of translation and results in the change in protein expression level. Especially, RNA G-quadruplexes present multiple functions in neurodegenerative diseases, affecting not only RNA–RNA interactions, but also RNA–protein interactions and altered the formation of membrane-less granules and RNA foci. The investigation of DNA/RNA G-quadruplexes in repeat expansion disorders will be helpful for our understanding of disease mechanism and the design of novel therapeutic strategies. In this review, G-quadruplex-forming repeats in neurodegeneration were summarized to reveal their roles in disease occurrence and development, and the potential of G-quadruplex binding ligands and proteins in the therapy of repeat expansion disorders was discussed.

## 2. Repeat Expansion Disorders

In the past decades, multiple repeats have been confirmed to be the genetic causes of neurodegeneration diseases. The association between repeat expansions and neurodegeneration diseases was first reported in 1991. A long CGG repeat located in FMR-1 gene was involved in the phenotypic expression of the fragile X syndrome [25]. Soon after, another report was published, declaring that the CAG repeat enlargement in androgen receptor gene was the potential genetic cause of SBMA [13]. Since then, researchers have been devoted to revealing the role of repeat expansions in hereditary neurodegeneration diseases. Many repeat mutations were found to associate with neurodegeneration, which were listed in Table 1. The disease-related repeat expansions could appear in the exon, the intron, the 5′ and 3′ untranslated region (UTR). Usually, the different locations of repeat expansions followed by different pathologies. In the diseases that repeat expansions occurred in the exons, abnormal poly amino acid stretches were introduced in the associated protein, resulting in gain-of-function mutations. To the contrary, repeats in the introns reduced the expression of mRNAs, leading to a loss-of-function mutation. In addition, the repeat expansions in non-coding regions, including introns and UTRs, also followed a gain-of-function of repeat RNAs, which could form toxic foci in cells.

The non-canonical structures of these repeats are described as the main causes of expansion [26], attracting the attention of most researchers. Except canonical Watson-Crick duplex, many non-canonical structures have been demonstrated to form in repeat sequences, including hairpins in CNG repeats (N = A, T, C or G), triplexes in GAA repeats, G-quadruplexes in GGGGCC repeats, unwinding structures in ATTCT repeats, and so on. Interestingly, multiple structures might exist in the same repeat sequences. For example, hairpin structures prevalently exist in CNG repeats, which are composed of C–G base pair and N-N mismatch [10,27]. In the presence of K+, CGG repeats were found to form intramolecular quadruplex [10,28]. Moreover, a special quartet structure, CGCG, was also observed in CGG repeats [29]. The diversity of secondary structures in repeat sequences increase the complexity in pathological research. Notably, G-quadruplex as one of the most studied non-canonical structures, is a threat factor to neurodegeneration. Therefore, deep understanding of G-quadruplexes functions in repeat expansions is significant for the investigation of disease mechanism and the development of novel therapy.

**Table 1:** A summary of repeat sequences existed in repeat expansion disorders.

Repeat Sequences	Locations	Related Diseases ^1^	Potential of G-Quadruplex Formation ^2^	References
CAG	extron, 5′ UTR	HD, SCA1, SCA2, SCA3, SCA6, SCA7, SCA12, SCA17, SBMA, DRPLA	No	[5,7,8,13,15,30,31,32,33,34,35,36]
CTG	extron, 5′ UTR, 3′UTR	SCA8, DM1,HDL2	No	[14,21,37,38]
CGG	5′ UTR	FXTAS, FXS	Yes	[25,39]
GAA	intron	FRDA	No	[40]
CCTG	intron	DM2	Possibly	[41]
ATTCT	intron	SCA10	No	[42]
TGGAA	intron	SCA31	Possibly	[43]
AAGGG	intron	CANVAS	Possibly	[44,45]
TTTCA	intron	BAFME1, BAFME4	No	[46,47]
GGGGCC	intron	ALS, FTD, PD	Yes	[11,12,19]
GGCCTG	intron	SCA36	Yes	[48]

^1^ The abbreviations of diseases in the table above: SCA, spinocerebellar ataxia; DM, myotonic dystrophy; FXTAS, fragile X tremor ataxia syndrome; FXS, fragile X syndrome; HD, Huntington’s disease; SBMA, spinal and bulbar muscular atrophy; DRPLA, dentatorubral-pallidoluysian atrophy; HDL2, Huntington’s disease-like 2; FRDA, Friedreich’s ataxia; ALS, amyotrophic lateral sclerosis; FTD, frontotemporal dementia; PD, Parkinson’s disease; CANVAS, cerebellar ataxia, neuropathy, vestibular areflexia syndrome; BAFME, benign adult familial myoclonic epilepsy. ^2^ The possibility of G-quadruplex formation in related repeat sequences. Yes—there is experimental evidence for G-quadruplex formation. Possibly—the repeats or their complementary strands may form G-quadruplex because their sequences conform to the form of (G_2–4_N_1–7_)_4_. No—no evidence is found for G-quadruplex formation.

## 3. G-Quadruplex-Forming Repeats in Neurodegeneration

G-quadruplex is one of the most frequently mentioned non-canonical structures in G-rich repeat expansions. Table 1 showed the potential G-quadruplex formation in repeat sequences. Three typical repeat expansions, CGG, GGGGCC, and GGCCTG, were introduced with experimental evidence. Except the three repeat sequences above, there were still many repeats with high G/C content, such as AAGGG [49], TGGAA [43], CCTG, and so on [50], which themselves, or via their complementary chains, may potentially form G-quadruplexes. Their secondary structures have not been fully characterized yet, and it is expected that future research could bring us more significant details.

### 3.1. CGG Repeats

CGG repeat expansion in 5′ UTR of FMR1 gene is the genetic cause of fragile X syndrome (FXS) and fragile X-associated tremor/ataxia syndrome (FXTAS) [25]. The number of CGG repeats in normal people is less than 50, while in the patients of FXS and FXTAS, the number of CGG repeats can be expanded to more than 200 [51]. As we have mentioned above, CGG repeat can adopt multiple structures, including homoduplex, hairpin, and tetraplex [52]. Fry demonstrated that DNA CGG repeats formed stable quadruplex structures in the presence of monovalent cations by electrophoretic analysis [53]. Patel’s work showed NMR evidence, which showed that G-quadruplex with a GCGC-tetrad formed in a sequence with CGG element, expanding the quadruplex folding topology [29]. However, later research found that FXS triplet repeat preferred hairpin structure moreso than tetraplex, suggesting that the folding of CGG repeats might greatly affected by repeat length and environmental condition [52]. Similar results were also reported for CGG RNAs. In the analysis of triplet repeat RNAs, all CNG repeats formed stable hairpin structures, and CGG was the most stable one [54]. Gdaniec’s group observed the formation of RNA G-quadruplex of two-repeat CGG sequence only in the presence of potassium, which was further characterized by ultraviolet-visible (UV), circular dichroism (CD), nuclear magnetic resonance (NMR) spectroscopies, and electrospray ionization mass spectrometry (ESI-MS) [55]. Binas reported that CGG-repeat-containing RNA present an equilibrium between a hairpin and a G-quadruplex structure, which was dependent on salt concentration and temperature [56]. Moreover, within CGG repeats, researchers found AGG interruption played an important role [57]. AGG repeats were demonstrated to form stable parallel G-quadruplex [54]. The presence of AGG interruption destabilized the stem–loop structures of CGG repeats, resulting in the inhibition of CGG expansion during replication and repair [57].

### 3.2. GGGGCC Repeats

In 2011, two independent groups published two individual works in Neuron, revealing the relationship between ALS/FTD and the GGGGCC repeat expansions in the intron of C9orf72 gene [11,12]. Later, this mutation was demonstrated to be the most common genetic cause of familial ALS/FTD, which was also detected in 5–20% of sporadic cases [58]. The GGGGCC repeats were soon proved to form hairpin structures and antiparallel G-quadruplex structures in the presence of K^+^, which were characterized by both CD spectrum and native polyacrylamide gel electrophoresis [59]. In further structural characterization, GGGGCC repeats were found to adopt different G-quadruplex topologies, which were highly dependent on not only the sequence and repeat numbers, but also the presence of monovalent and bivalent cations [60,61]. As with DNA repeats, GGGGCC RNA repeats were demonstrated to form stable unimolecular and multimolecular parallel G-quadruplexes, which were greatly affected by repeat number and RNA concentration [62]. Zhang et al. published the results of molecular dynamics simulations of GGGGCC DNA and RNA repeats to characterize the local conformation of G-quadruplexes and the ion distribution, expanding the understanding of GGGGCC repeat structures at the atomic level [63]. Moreover, researchers found that CD and NMR evidence showed the complementary antisense sequences, as well as GGCCCC repeats, forming i-motif and protonated hairpin structures under near-physiological conditions, even in the presence of the sense strand [64]. A report declared that the antisense GGCCCC repeats formed antiparallel quadruplex composed of GCGC-tetrads and CCCC-tetrads, similar to a folded hairpin, and the methylation of CpG could alter the structures in both sense and antisense strands that further potentially affect epigenetic processes [65].

### 3.3. GGCCTG Repeats

SCAs are a group of neurodegenerative disorders, which have diverse mutation types [66]. In 2011, Japanese researchers found that GGCCTG repeat expansion in intron 1 of NOP56 was the genetic cause of SCA36 [48]. Zhang et al. reported the G-quadruplex models of GGGCCT repeats by molecular dynamics simulations [63]. G-quadruplexes formed by GGGCCT repeats were less stable compared to GGGGCC repeats due to the lack of a layer of G-quartet stacking, and the antiparallel models were more unstable than the parallel model. Recently, the Ikeda group demonstrated the formation of G-quadruplex in GGCCTG repeats by CD, and the colocalization of G-quadruplex antibody and RNA foci formed by transcribed GGCCTG RNA repeats further confirmed the presence of RNA G-quadruplexes in GGCCTG repeats [67]. Yi and coworkers characterized the diverse structures of GGCCTG repeats by NMR, including hairpin, duplex, and G-quadruplex, and the formation of antiparallel G-quadruplex was observed in (GGCCTG)_2_ [68].

## 4. Functions of G-Quadruplexes in Neurodegeneration

**Figure 1:**
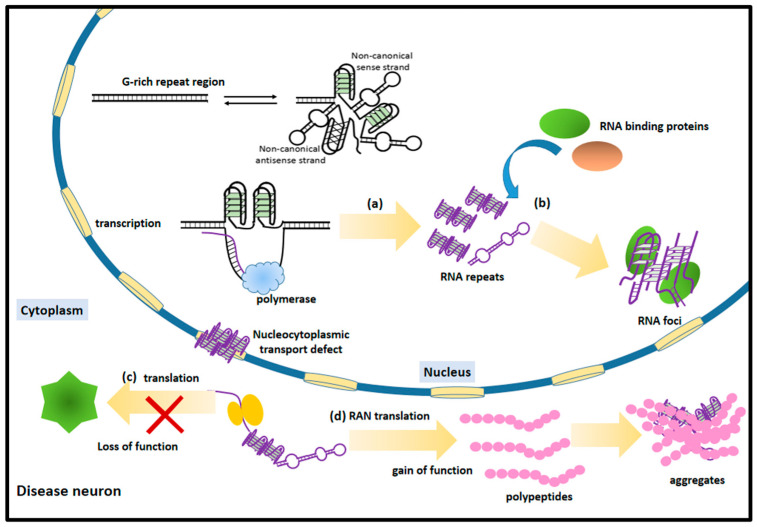
Processes that affected by G-quadruplex formation in repeat expansion disorders. (**a**) The formation of G-quadruplexes during transcription resulted in aborted transcripts and repeated RNAs. (**b**) G-quadruplexes in RNA repeats facilitated the formation of RNA foci and affected the interaction between RNA and RNA binding proteins. (**c**) The formation of G-quadruplexes inhibited the normal translation, resulting in the loss of important proteins. (**d**) The RAN translation in repeat expansion led to the production of toxic polypeptides and further aggregated with RNA structures.

Researchers have been working on the mechanism of repeat expansion disorders in recent years. Three main mechanisms have been proposed: the accumulation of toxic RNAs with repeat sequences; the loss-of-function of corresponding proteins; the gain-of-function peptides and proteins transcribed from repeat-containing RNAs [58]. In neurodegeneration diseases caused by G-rich repeat expansion, each of them is related to the formation of G-quadruplexes in the repeat sequences. Figure 1 showed the processes which might be affected by G-quadruplex formation in repeat expansion disorders. The structure and location of repeat sequences are crucial factors for our understanding of pathogenic mechanisms.

### 4.1. Variable Repeat Length in Replication and Regulation of Transcription

The sizes of repeat expansions vary in a large range from dozens to thousands, which potentially correlates with the symptoms of neurodegeneration diseases, such as the onset age, risk, and the severity of diseases [24]. The repeat expansions are described as a dynamic process where the lengths continue to change within tissues and generations due to the gene instability. The repeat expansions with multifarious structures obviously increase the gene instability, which further affects processes, including replication, repair, transcription, and translation. G-quadruplex structures have been demonstrated to induce genomic instability and DNA damage [69]. The G-quadruplexes formed in repeat regions are considered to play important roles in these biological processes.

In the replication of repeat sequences, the formation of non-canonical structure results in the expansion or contraction [70]. During replication, the duplex template unwinded temporarily, providing a condition for non-B structure formation in both template strand and nascent strand. The variation of repeat lengths in inherited trinucleotide repeat disorders have been studied for years, and excellent reviews have explained the mechanisms of dynamic mutations with repeat induced gene instability in detail [71,72]. Especially, in the replication of CGG repeats, the unusual secondary structure of G-rich lagging strand-like hairpin and G-quadruplex resulted in intra-array deletions, suggesting relevance to the repeat expansion in humans [73,74]. Similar replication dynamics were also observed in the replication of GGGGCC repeats [75]. G-quadruplex-forming sequences in the nascent strand resulted in repeat expansion, whereas G-quadruplex-forming sequences in the lagging strand caused repeat contraction. The replication was perturbed by GGGGCC repeat expansion with a decreased overall efficiency and an increased instability. It was highly dependent on the length of GGGGCC repeat, which might be due to the fact that longer repeats can form more stable G-quadruplex structures.

The formation of G-quadruplex structures in repeat expansions also greatly affect the process of transcription. One feature of repeat expansions is the bidirectional transcription, generating both sense and antisense RNA products [68,69,70]. The formation of double R-loops induced by bidirectional transcription has been demonstrated as one of the reasons for the genetic instability [71]. R-loop is the DNA:RNA hybrid, forming temporarily during the transcription. R-loop was demonstrated to inhibit the transcription, to promote antisense RNA transcription, and to inhibit DNA methylation at the promoter [72]. In repeat expansion region, the transcription is usually bidirectional, and R-loops formed both in sense and antisense strands, which bring greater instability than a single R-loop. Interestingly, the transcription across G-rich repeats resulted in more R-loops compared to the transcription across C-rich repeats, which were both observed in the transcription of GGGGCC/GGCCCC repeats and CGG/CCG repeats [76]. The possible explanation was that R-loops were stabilized by the G-quadruplex formation in non-transcribed G-rich DNA repeat strands or the G-quadruplex formation between DNA repeats and RNA transcripts. On the other hand, G-rich repeats were usually located in the non-coding regions, such as UTRs, introns, and promoters, and the formation of G-quadruplex in these regions is crucial for the regulation of transcription [77]. It was reported that G-quadruplex in the template strand could significantly inhibit the transcription, resulting in pause and arrest during transcription [78]. Haeusler et al. found that the formation of G-quadruplexes in GGGGCC repeats impaired the transcription, resulting in aborted transcripts and a decrease in full-length transcripts [59].

### 4.2. Accumulation of Toxic RNA Repeats

Most repeat expansion disorders suffer from the formation of toxic RNA foci regardless of the related repeats located in exons, introns, or UTRs. The RNA foci formed by repeat expansions in nucleus was observed in both cells and tissues. Generally, the formation of RNA foci increased the stress of nucleus [79,80], sequestered RNA binding proteins [81], and impaired the nucleocytoplasmic transport [79], ultimately resulting in different neurodegeneration symptoms.

The secondary structures play important roles in the accumulation of RNA repeats. Similar to homologous DNA repeat, RNA repeats also form various non-canonical structures, including hairpin structures, triplexes, and tetraplexes. For example, the RNA repeats of CMG (M = A, U, C or G) also formed hairpins with a stem composed of C–G base pairing and non-canonical M–M interaction [54]. Based on the characterization with biochemical and biophysical methods, CMG repeats were demonstrated to form stable hairpins, and CGG repeats were most stable among them [54]. The repeats of GGGGCC showed mixed structures with parallel G-quadruplex and hairpin [59]. Kiliszek has made a comprehensive overview concerning the structures of RNA repeats, which are associated with neurological diseases in great detail and summarized the secondary structures in present probing and crystallographic studies [82]. These non-canonical structures of RNA repeats stacked onto each other and resulted in the formation of insoluble RNA foci.

In 2017, CAG repeats and GGGGCC repeats were observed to form RNA gels by phase transitions [83]. The RNA gelation happened with critical repeat numbers and the presence of polyvalent cations, and the RNAs in the clusters are immobile. The authors hypothesized that RNAs initially phase-separated into spherical liquid-like droplets and rapidly became cross-linked into gels through intermolecular base-pairing. In addition, CAG repeats showed liquid-like characteristics, while GGGGCC repeats displayed gel-like properties, which might be explained by the different intermolecular interactions. In GGGGCC repeats, besides intermolecular hairpin structure, intermolecular G-quadruplex structure was also an important part in RNA gelation. These results provided evidence that toxic sequence-specific RNA gels should be a contributing factor in the study of neurological diseases. Moreover, in the comparison, GGGGCC exhibited less dynamic characteristics than CAG RNA foci, suggesting a stronger intermolecular interaction by intermolecular G-quadruplex formation.

The secondary structures and intermolecular interactions of RNA repeats during RNA gelation have shown their importance in the pathological study of neurodegeneration. Zhang et al. found G-quadruplex structure triggered RNA phase separation, emphasizing the importance of G-quadruplex formation in RNA-driven phase separation [84]. Similar to their results, the formation of G-quadruplex was demonstrated to facilitate the RNA accumulation in the comparison of CAG, CUG, GGGGCC, and GGGTTA repeats, especially under the molecular crowding condition [85]. Further research indicated that the level of RNA gelation was related to the dielectric constant of the solution, suggesting that electrostatic interactions were the main factor leading to RNA phase separation.

### 4.3. Loss/Gain of Function of Proteins and Peptides

As the crucial roles that almost involved in all life processes, peptides and proteins assumed many functions within organisms. In repeat-associated neurodegeneration diseases, the abnormalities in peptides and proteins are critical parts in pathological study. Repeat expansions have been demonstrated to affect the expression of proteins. The loss/gain of function of proteins and peptides was described as another main pathogenesis of repeat expansion disorders, which showed different features according to the position of repeat expansion in disease-related gene [50,86,87,88,89].

The loss of function of proteins is usually the result of the downregulation or inhibition of related gene expression caused by repeat expansions. Non-canonical structures have been demonstrated to efficiently affect the processes of transcription and translation in previous reports [78,90,91,92]. The formation of G-quadruplexes in DNA templates caused pause, slippage, and arrest in transcription, potentially reducing the production of normal mRNAs [78]. The splicing of RNAs and translation of mRNAs are also regulated by the formation of non-canonical structures in RNA [90,91,93,94,95]. In the cases of repeat expansion disorders, the expression of proteins also followed the influence of tetraplexes. As a result, the production of mRNAs and wild-type proteins were usually suppressed. For instance, in C9orf72-related ALS/FTD caused by GGGGCC repeat expansions, three variants of transcripts were generated from the transcription. All three mRNA variants in patient samples had different degrees of reduction, suggesting a loss of function mechanism [11,96].

The gain of function of peptides and protein is another major pathogenesis of repeat expansion disorders. The repeat-associated non-ATG translation (RAN translation) is discovered to occur without an ATG start codon, leading to the production of polypeptides that are potentially toxic to neurons [97]. The detailed mechanism of this repeat-associated unconventional translation is still not fully understood. Some research works suggested that the RAN translation might follow different mechanisms in different repeat types. Todd has summarized the information and problems about how RAN translation initiated and highlighted the recent finding in repeat expansions that related to neurodegeneration diseases [98]. The RAN translation started from a near-homologous start codon CUG located upstream of the repeat and generated homopolymeric peptides with polyglycine and polyalanine in CGG repeat expansions and poly-dipeptides, such as poly-GR, poly-GA, and poly-GP in GGGGCC repeat expansions. These peptides induced neural toxicities, and numerous reports were published discussing their effects on many bioprocesses [88]. They were high molecular weight and insoluble in brain homogenates and neuronal inclusions, but not in peripheral tissues [99]. They impair in many cellular processes [100], including nucleolar function [80], nucleocytoplasmic transport [101], RNA splicing [102], and global translation [103], but the mechanisms of their neurotoxicity were still unclear. Many researchers are working on revealing the pathogenesis and developing efficient therapeutic methods. The secondary structure of RNA plays an important role in RAN translation. The initiation of RAN translation was greatly affected by repeat length, reflecting the importance of their secondary structure, though it was still unclear about whether the exact structures were hairpins or G-quadruplexes [104]. GGGGCC RNA repeats adopted both hairpin and G-quadruplex structures, which possibly trigger the RAN translation [105]. Recently, Wang’s group reported that RNA helicase DHX36 selectively bound and winded G-quadruplexes in GGGGCC repeats and facilitated the RAN translation, suggested that G-quadruplex in repeat expansion might be a potential target to regulate the production of toxic peptides [106].

Moreover, the aggregation of peptides and proteins was also affected by the presence and structure of RNA through phase separation. In 2018, researchers have demonstrated the effects of RNA on the phase separation of RNA-binding proteins from different points of view. Maharana et al. found the solubility of prion-like RNA binding proteins, such as TDP-43 and FUS, regulated by RNA concentration [107]. The reduction of nuclear RNA level caused toxic solid-like aggregations. Gladfelter’s group revealed that the structures of mRNA and RNA-RNA interaction determined the identity of droplets in the phase separation driven by polyQ-protein Whi3 [108]. Since then, researchers began to pay more attention to the regulatory role of RNA structure in phase separation. Navarro et al. demonstrated that RNA acted as the molecular seed in the condensation nucleation, affecting the composition and morphology of RNA–protein assemblies [109]. In addition, the highly structured RNA was proved to rearrange protein aggregates, suggesting the importance of RNA structure in protein phase separation [110]. Recently, G-quadruplex formed by CGG repeats were demonstrated to interact with the toxic protein FMRpolyG produced by RAN translation and to induce neuronal dysfunction [111]. More works about G-quadruplex in the regulation of protein aggregation are expected in the future.

## 5. Targeting G-Quadruplexes for Therapy

The formation of G-quadruplex in repeat sequence is important for our understanding of repeat expansion disorders, which are highly involved in many pathogenic processes, such as repeat expansion, regulation of gene expression, the accumulation of RNA and peptides. Therefore, targeting on G-quadruplex provide a new perspective for pathological research and the development of new therapeutic methods. Small molecule G-quadruplex binding ligands and G-quadruplex binding proteins are frequently utilized for targeting G-quadruplex.

Small molecules, which can selectively bind to G-quadruplexes, have been widely studied in anticancer and antiviral strategies [112,113,114,115]. Simone and coworkers screened for three small molecules specifically stabilized G-quadruplex. They were demonstrated to reduce the RNA foci formed in C9orf72 ALS/FTD and decrease the expression level of toxic poly-dipeptides [116]. A widely used G-quadruplex ligand, TMPyP4, was found to modulate the G-quadruplex formed by GGGGCC RNA repeat and disrupt the interaction between G-quadruplex and proteins such as hnRNPA1 and ASF/SF2, suggesting the importance of G-quadruplex in protein sequestration and RAN translation [117]. TMPyP4 and porphyrin derivatives including sodium copper chlorophyllin and hemin chloride were also observed to reduce the cytotoxicity of GGCCUG repeat expansion because of the interaction with G-quadruplex formed in GGCCUG repeats [67]. Porphyrins protoporphyrin IX (PpIX) was discovered to interact with G-quadruplex structures formed in CGG repeats, resulting in the reduction of RAN translation and inhibition of the interactions between CGG G-quadruplex and FMRpolyG proteins, thereby presenting the amelioration of neuronal dysfunction [111]. Moreover, there are also some reports about small molecules targeting the hairpin structures formed in G-rich repeats. Disney’s work reported small molecules, which could recognize the hairpin structures over G-quadruplexes in GGGGCC repeats and CGG repeats, inhibiting the formation of RNA foci and RAN translation [118,119,120]. Murase et al. investigated a selective small molecule targeting G–G mismatch in the hairpin formed by CGG repeat to converse the G-quadruplex into hairpin [121]. This research explained the importance of G-quadruplex in the pathological study of repeat expansion disorders from another side.

G-quadruplex binding proteins are attractive research objects to target G-quadruplexes because they are involved in many biological processes. The interaction between G-quadruplex and related G-quadruplex binding proteins are crucial for pathogenic mechanisms of repeat expansion disorders. For example, nucleolin was found to specifically bind to G-quadruplex formed by GGGGCC repeats, resulting in nucleolar stress [59]. Helicases, which could unfold the G-quadruplex, have been studied for years. As previously mentioned, the RNA helicase DHX36 has been demonstrated to selectively bind to G-quadruplex formed by GGGGCC RNA repeats and to facilitate the RAN translation [106]. Tseng et al. also reported that DHX36 could improve the efficiency and completion in the transcription of GGGGCC repeats, as well as the RAN translation of GGGGCC repeats and CGG repeats impaired by DHX36 depletion [122]. On the contrary, RNA helicase DDX3X was found to bind GGGGCC repeats, rather than the G-quadruplex structure formed by the repeats [123]. DDX3X suppressed RAN translation of GGGGCC repeats and decreased the expression level of poly-dipeptides, further illustrating the potential of targeting the diverse structures of repeat sequences in neurodegeneration therapy. Moreover, Pura protein can bind to G-rich repeat, such as GGGGCC and CGG. A Pur-based peptide, TZIP, was designed to bind both G-quadruplex and linear repeat RNAs. It was demonstrated to unwind G-quadruplex and convert it to aggregated higher-order structures [124]. It might further modulate the interaction between GGGGCC G-quadruplex and other proteins, providing new targets for therapy.

In addition to the two strategies above, antisense oligonucleotides (ASO) and gene editing with CRISPR/Cas9 system are also promising therapeutic approaches of repeat expansion disorders. Short complementary sequences were observed to reduce the formation of RNA foci by disrupting the base pairing in the assembly process [83]. Liu and coworkers found stereopure oligonucleotides that selectively reduced the production of repeat-containing transcripts in mouse models and protected neurons from glutamate-induced toxicity [125]. ASOs were also reported to be modified to obtain high target affinity and high nuclease resistant to reduce RNA foci in GGCCTG repeat expansion without altering NOP56 mRNA expression levels [126]. On the other hand, CRISPR/Cas9 system is an attractive tool that could excise the repeat expansions from the related genome with the presence of designed gRNAs. There have been several reports about the applications of CRISPR/Cas9 system in repeat expansion disorders [127,128]. For example, the excision of GGGGCC repeat expansion in ALS/FTD resulted in the reduction of RNA foci and poly-dipeptides, suggesting the potential in neurodegeneration therapeutics [128]. These methods are not directly targeting on G-quadruplexes in repeat expansion, but possibly modulate G-quadruplex conformations and protein–G-quadruplex interactions.

## 6. Discussion

The neurodegeneration diseases that are caused by repeat expansions have been studied for more than 20 years since the first discovery of the relationship between long CGG repeats and FXS in 1991. Though there are still many problems that remained unsolved, we have gained a lot of significant information associated with pathology and therapy. The neurodegeneration diseases associated with repeat expansions have many similar characters and processes, but they also show their own specific mechanisms and pathologies. These studies involve many scientific fields in chemistry, biology, and medicine, and they still need a long way to go. The formation of G-quadruplex is demonstrated to be an important factor in neurodegeneration with G-rich repeat expansion, associated with repeat expansion in replication, reduction of mRNAs in transcription, formation of toxic RNA foci, and production of toxic polypeptides by RAN translation. Targeting on the G-quadruplex in repeat expansion is a significant strategy to protect neurons from dysfunction and delay the progression of neurodegeneration. More evidence and information are expected to provide insight into disease mechanisms and to achieve successful therapeutic methods.

Currently, problems remain in the explanation of disease mechanisms. For example, researchers still have some doubts about the relationship between repeat lengths and neurodegeneration. In one of the initial studies of the relationship between repeat expansions in C9orf72 and ALS/FTD, though the comparison of ALS/FTD patients and controls showed an obvious difference in the region where repeat numbers are more than 30, there are still 38.1% cases carried the same repeat expansion as healthy people. In a recent stdy, the authors summarized 49 identified studies of C9orf72-related ALS/FTD and found that the lengths of C9orf72 hexanucleotide repeats were associated not with a higher risk of disorders, but a higher frequency of neuropsychiatric symptoms [129]. In addition, due to the limitation of the repeat-primed PCR, the actual need of repeat length to cause diseases remains unknown. Though a repeat length of over 30 is generally accepted to cause the neurodegeneration in C9orf72-related ALS/FTD sufficiently, smaller repeat sizes, such as 20–30, have also been reported to be pathogenic [130,131]. A more extensive mechanism might be required in the pathological study of repeat expansion disorders. Interestingly, researchers found that, in the formation of GGGGCC gels, the repeat number for RNA gelation in cell condition is higher than that in vitro. This suggests that the cellular environment plays an important role in the formation of RNA gels. Moreover, in experiments with mice, researchers found that the expression of RNA repeats and polypeptides did not result in the symptoms of neurodegeneration [125]. These results suggested the presence of other important factors, such as environmental conditions.

According to our knowledge, the structures of nucleic acids are greatly affected by the surrounding environments, especially the non-canonical structures, such as G-quadruplex under crowded condition. Researchers previously reported various changes in neurological functional biomolecules in cellular environments. For example, in the serum and spinal fluid of patients, a higher level of glutamate, the main excitatory neurotransmitter, was observed [132]. There are also reports that cations in cells also changed in ALS/FTD cases. An increased concentration of Ca2+ was found in patients’ cells. A global loss of Na, as well as K-ATPase, resulted in the dysfunction in the sodium/potassium ion pump [133]. These changes are all potentially affect G-quadruplex folding and regulate the gene expression. In addition, the nucleocytoplasmic transports are always defected in neurodegeneration diseases [102,134,135], which potentially causes the mislocalization and cytoplasmic aggregation of proteins, perturbing the metabolism of RNAs and proteins and changing cellular environments [136]. Moreover, the production and accumulation of misfolded proteins and peptides, which have been demonstrated, can be transmitted cell-to-cell through exosomes, which can also change the cell conditions [137]. The effects of cations, small biomolecules, and non-toxic peptides have not attracted many attentions yet. Considering the crucial environmental effects on the structures and interaction of nucleic acids, further research about environmental effects on G-quadruplex-forming repeats is expected in the future.

## Data Availability

No new data were created or analyzed in this study. Data sharing is not applicable to this article.
